# Factors Influencing the Diversity of Iron Uptake Systems in Aquatic Microorganisms

**DOI:** 10.3389/fmicb.2012.00362

**Published:** 2012-10-18

**Authors:** Dhwani K. Desai, Falguni D. Desai, Julie LaRoche

**Affiliations:** ^1^Biological Oceanography Division, Helmholtz-Zentrum für Ozeanforschung Kiel (GEOMAR)Kiel, Germany

**Keywords:** marine microbes, eukaryotic phytoplankton, Fe limitation, Fe- binding ligands, multivariate statistics, metagenomes, dN/dS ratio, aquatic niches

## Abstract

Iron (Fe) is an essential micronutrient for many processes in all living cells. Dissolved Fe (dFe) concentrations in the ocean are of the order of a few nM, and Fe is often a factor limiting primary production. Bioavailability of Fe in aquatic environments is believed to be primarily controlled through chelation by Fe-binding ligands. Marine microbes have evolved different mechanisms to cope with the scarcity of bioavailable dFe. Gradients in dFe concentrations and diversity of the Fe-ligand pool from coastal to open ocean waters have presumably imposed selection pressures that should be reflected in the genomes of microbial communities inhabiting the pelagic realm. We applied a hidden Markov model (HMM)-based search for proteins related to cellular iron metabolism, and in particular those involved in Fe uptake mechanisms in 164 microbial genomes belonging to diverse taxa and occupying different aquatic niches. A multivariate statistical approach demonstrated that in phototrophic organisms, there is a clear influence of the ecological niche on the diversity of Fe uptake systems. Extending the analyses to the metagenome database from the Global Ocean Sampling expedition, we demonstrated that the Fe uptake and homeostasis mechanisms differed significantly across marine niches defined by temperatures and dFe concentrations, and that this difference was linked to the distribution of microbial taxa in these niches. Using the dN/dS ratios (which signify the rate of non-synonymous mutations) of the nucleotide sequences, we identified that genes encoding for TonB, Ferritin, Ferric reductase, IdiA, ZupT, and Fe^2+^ transport proteins FeoA and FeoB were evolving at a faster rate (positive selection pressure) while genes encoding ferrisiderophore, heme and Vitamin B12 uptake systems, siderophore biosynthesis, and IsiA and IsiB were under purifying selection pressure (evolving slowly).

## Introduction

Iron-containing metalloenzymes are essential for many life processes, including photosynthesis, respiration, and nitrogen fixation. Due to the tendency of Fe^3+^ to form ferric hydroxides and oxyhydroxide polymers in the presence of oxygen, the dFe concentration in surface seawater is <0.5 nM (Johnson et al., 1997). Aeolian dust deposition is the dominant external source of iron in the open ocean surface waters (Duce and Tindale, 1991; Jickells et al., 2005) of the North Atlantic and North-East Pacific accounting for 48 and 22% of total Fe deposition respectively (Gao et al., 2001). In around 40% of the world’s oceans where surface waters are high in nutrient and low in chlorophyll (HNLC regions), low Fe supply limits the growth of resident eukaryotic phytoplankton and cyanobacteria responsible for primary production (Martin et al., 1994; Coale et al., 1996; Boyd et al., 2000; Gall et al., 2001; Tsuda et al., 2003; de Baar et al., 2005).

Diverse strategies have evolved to competitively acquire enough iron for survival in various oceanic habitats. This competition is intensified by the fact that >99.9% of dFe is complexed to Fe-binding ligands of diverse nature (Gledhill and van den Berg, 1994; Rue and Bruland, 1995; Gledhill and Buck, 2012). Thus, microorganisms have developed systems to take up Fe from a wide range of Fe-binding ligands. Although ubiquitous in nature, Fe-binding ligands vary in their Fe-binding affinities and their distribution from surface to deep waters (Rue and Bruland, 1995; Hunter and Boyd, 2007) and from coastal to open ocean waters (Boye et al., 2003; Buck and Bruland, 2007).

The ability to produce siderophores in the open ocean is apparently, largely confined to heterotrophic bacteria (Reid et al., 1993; Martinez et al., 2000; Butler, 2005; Martinez and Butler, 2007; Homann et al., 2009a,b). While siderophore biosynthesis pathways have been found in some coastal (Ito and Butler, 2005) or fresh water (Ito et al., 2004) cyanobacteria, they appear to be absent in open ocean cyanobacteria. Coastal strains of *Synechococcus* that have been reported to produce siderophores (Ito and Butler, 2005) have high Fe-quotas compared to oceanic strains (Palenik et al., 2006). Even though only a few marine microorganisms can synthesize siderophores, the ability to take up siderophores may be more widespread, as evidenced by the abundance of TonB-dependent (TBD) siderophore uptake systems observed in terrestrial and freshwater microorganisms devoid of siderophore synthesis pathways (Plessner et al., 1993; Katoh et al., 2001; Poole and McKay, 2003; Joshi et al., 2008). Additionally, various heme-acquisition systems have been identified in bacteria for the utilization of Fe bound to heme (Stojiljkovic and Hantke, 1994; Cope et al., 1995; Thompson et al., 1999; Ochsner et al., 2000; Hopkinson et al., 2008). Notably, siderophore biosynthesis and TBD siderophore/heme uptake receptors are absent from the genomes of *Prochlorococcus* and strains of *Synechococcus* that dominate the microbial communities of open ocean surface waters (Waterbury et al., 1979; Liu et al., 1997, 1999; Hopkinson and Morel, 2009). The reduction of cellular Fe requirements provides an alternate adaptive strategy for surviving Fe limitation in open ocean surface waters. This strategy has been observed in the *Prochlorococcus* ecotypes (Thompson et al., 2011) dominant in HNLC waters, which have decreased their Fe-quotas by eliminating several Fe-requiring proteins (Rusch et al., 2010).

The availability of a large number of marine prokaryotic and eukaryotic microbial genomes and the Global Ocean Sampling (GOS) metagenomes, have greatly enhanced our understanding of the Fe-acquisition strategies used by various groups of microorganisms. Recent studies by Hopkinson and Barbeau (2012) and Toulza et al. (2012) presented sequence-based approaches to analyze the differences in occurrence patterns of proteins involved in Fe-metabolism in marine prokaryotic genomes and metagenomes, respectively. Hopkinson and Barbeau (2012) reported a dominance of TBD uptake systems in *Gammaproteobacteria*, and identified a novel heme TBDT in *Prochlorococcus* which may have been acquired by horizontal gene transfer, providing niche specific adaptation in this organism. Their study further revealed the widespread occurrence of Fe^3+^ ABC transporters in all groups of marine bacteria except for Flavobacteria, and a lack of the specific Fe^2+^ uptake system (FeoAB) in picocyanobacteria and *Alphaproteobacteria*. They identified that the TBDTs were less common in the metagenomes than in the genomes, an observation that reflects the numerical dominance of *Pelagibacter* and *Prochlorococcus* (Rusch et al., 2007) in the current collection of GOS metagenomes. Both genera have small genomes and non-specialized iron uptake systems (Smith et al., 2010; Thompson et al., 2011). A more detailed analysis of the Fe-metabolism proteins in the GOS metagenomes revealed a distribution pattern influenced by dFe concentrations (Toulza et al., 2012). The frequencies of occurrence of Fe^3+^ transporters and of Fe^2+^ uptake systems were negatively correlated with each other, the former being more abundant in the open ocean environments and the latter in the coastal environments, respectively. The taxonomic diversity and Fe-pathway prevalence differed significantly with habitat or niche type (Open Ocean or Coastal). However the GOS samples are distributed along a wide range of latitudes possibly confounding the effect of temperature, which varies widely within both Open Ocean and Coastal niches. In addition, prior studies did not directly investigate the exact nature of the link between taxonomic diversity and Fe-pathway prevalence within the metagenomes.

Here, we build on the results presented in the studies of Hopkinson and Barbeau (2012) and Toulza et al. (2012), by extending the analyses to marine eukaryotic genomes of phytoplankton. We established a link between the Fe-metabolism profiles and taxonomic diversity prevalent in the metagenomes by comparing the Fe-metabolism protein occurrence profiles (Table 1) from the genomes of various taxa and those obtained from various aquatic niches defined by environmental characterization of metagenomes. Marine prokaryotic and eukaryotic microorganisms were grouped according to their location of isolation (Open Ocean, Coastal, or Freshwater) to check for niche specific adaptations reflected in their genomes. The GOS metagenomes were subjected to a more complex grouping in order to account for differences in Fe-metabolism protein profiles of the microbial community, which could be attributed to temperature, dFe concentration, or a coastal versus Open Ocean sampling location. Thus, we defined three contrasting niche group pairs in the GOS metagenomes such that the groups of samples in a pair differed with respect to only one of the above environmental factors. A multivariate statistical approach was used to study the differential distribution of Fe-metabolism protein profiles in genomes and in the above GOS metagenomic groupings to establish whether the Fe-metabolism strategies were correlated with the taxonomic distribution in the GOS metagenomes. We found a set of proteins that were statistically discriminating between the aquatic niches. Calculations of the non-synonymous mutation rates (dN/dS) for this set of proteins indicated that they were under positive selection pressure and therefore were evolving rapidly.

**Table 1:** **List of proteins involved in Fe-ligand (siderophore and heme) uptake, siderophore, and heme biosynthesis and Fe homeostasis in microorganisms**.

System	Abbreviation	Genetic nomenclature	Reference
Heme direct uptake	Heme-Upt	PhuRSTUVW (*Pseudomonas aeruginosa*), HemRSTUV (*Yersinia enterocolitica*), HutABCD, HutR (*Vibrio cholerae*), BhuRSTUV (*Bordetella pertussis*), HmuRSTUV (*Y. pestis*)	Stojiljkovic and Hantke (1994), Thompson et al. (1999), Ochsner et al. (2000), Mey and Payne (2001), Vanderpool and Armstrong (2003)
Hemophore-mediated heme uptake		HasRADEF (*Pseudomonas aeruginosa*)	Lewis et al. (1997), Ochsner et al. (2000)
Heme uptake through bipartite receptors		HpuAB (*Neisseria* sp.)	Lewis et al. (1997)
Hydroxamate siderophore uptake	Hydrox	FhuABCD (ferrichrome), FhuE (rhodotorulic acid), IutA (aerobactin) and FoxA (ferrioxamine B) in *E. coli* K-12, FcuA (ferrichrome) in *Yersinia enterocolitica*, FegAB in *Bradyrhizobium japonicum* 61A152, RhtAX (rhizobactin) in *Sinorhizobium meliloti*, PupA and PupB pseudobactin receptors in *Pseudomonas putida*	Fecker and Braun (1983), Koebnik et al. (1993b), Koster et al. (1995), Lynch et al. (2001), Braun (2003), Benson et al. (2005)
Catecholate siderophore uptake	Catech	FepABCD for enterobactin, BtuBFCD for vitamin B12 and colicin receptor CirA in *E. coli*, pesticin receptor FyuA in *Yersinia enterocolitica*, PfeA for ferric-enterobactin in *Pseudomonas*, vibriobactin receptor ViuA and enterobactin receptor IrgA in *Vibrio cholera*	Worsham and Konisky (1985), Butterton et al. (1992), Rakin et al. (1994), Cadieux et al. (2002), Braun (2003), Cornelis and Bodilis (2009)
Citrate siderophore uptake	Citrate	FecABCD (citrate) in *Escherichia coli*	Braun (2003)
Ferric binding periplasmic protein dependent Fe3+ transporters	Fe3+	IdiA, HitABC, FbpA	Sanders et al. (1994), Adhikari et al. (1995), Ferreiros et al. (1999), Webb et al. (2001)
Fe2+ uptake or uptake of divalent cations	Fe2+	FeoAB, ZupT, MgtE, FTR1	Kammler et al. (1993), Guerinot (2000), Grass et al. (2005)
Energy coupling for TonB-dependent (TBD) ligand uptake	Ener Coup	TonB/ExbB/ExbD	Koebnik et al. (1993a)
Non-ribosomal peptide synthetase	NRPS	NRPS	Jeanjean et al. (2008)
NRPS independent siderophore synthesis	NIS	IucA, IucC – aerobactin; RhbB, RhbDF – rhizobactin; DesB, DesD – Desferrioxamine	Challis (2005)
Heme/chlorophyll biosynthesis	Hem-Syn	HemBCEF	Mochizuki et al. (2010)
Heme oxygenase	Hem-Oxy	HemO, HemS, HmuS, HO1, HmuO	Thompson et al. (1999)
Regulatory elements	Regul	Fur, DtxR, Rir	Wexler et al. (2003), Johnston et al. (2007)
Ferritin-like Fe storage	FeStor	Ferritin-dps, BfrAB	Andrews (1998)
Fe-stress induced homeostasis genes	IsiA	IsiA	Burnap et al. (1993)
Flavodoxin	IsiB	IsiB	LaRoche et al. (1996)
Ferric reductase	Fe-Red	Ferric reductase	Kosman (2003)

## Materials and Methods

### Hidden Markov model-ModE profiles of iron metabolism genes

The set of non-redundant (Uniref 100) protein sequences for the genes listed in Table 1 were downloaded from Uniprot and HMM-ModE profiles were created as described earlier (Srivastava et al., 2007). The HMM-ModE protocol allows the construction of HMM profiles with increased specificity by using negative training sequences.

The training sequences for each protein were first clustered using the Markov Clustering Algorithm (MCL) (Enright et al., 2002). For each subgroup of each protein, the training sequences were aligned with MUSCLE (Edgar, 2004) and HMMs were generated using *hmmbuild* from the HMMER2 package (Eddy, 1998). The discrimination threshold of each protein HMM was optimized by an *n*-fold cross-validation exercise. The training sequences for each were divided into *n* test sets such that each sequence is part of at least one test set. For each test set *t*, the remaining (*n*-1) sets were combined to form the train set and used to build an HMM. The sequences in *t* were scored using this HMM by *hmmsearch* program to get a True Positive (TP) score distribution. False positives (FP) were identified from the negative training set (in this case the entire UniProt database excluding the training sequences for the gene in question). The sensitivity, specificity, and Matthews Correlation Coefficient (MCC) distribution for each of *n* sets was calculated (Hannenhalli and Russell, 2000). The optimal discrimination threshold was identified as the mid-point of the MCC distribution averaged over the *n* sets. Further increase in specificity was obtained by modifying the emission probabilities of the gene HMM by using the FP alignment as described earlier (Srivastava et al., 2007).

These HMM-ModE profiles with their optimized threshold were used with the program *hmmsearch* to scan the protein sequences from the marine microbial genomes as well as from the GOS metagenomes. All the above steps were performed using customized Perl scripts that are available for download from https://sites.google.com/site/dhwanidesai/home/bioinformatics.

### Obtaining the complete genome sequences of marine microbes

Complete genome sequences of marine microbes whose sequencing projects were commissioned by the Gordon and Betty Moore Foundation under their Marine Microbiology Initiative^1^ were downloaded from NCBI in the form of protein FASTA files. Complete genomes of six eukaryotic marine microorganisms including *diatoms* (*Thalassiosira pseudonana*, *T. oceanica*, and *Phaeodactylum tricornutum*), a pelagophyte (*Aureococcus anophagefferens*), green algae (*Ostreococcus lucimarinus* and *Ostreococcus tauri*), and a prymnesiophyte (*Emiliania huxleyii*) were also downloaded from NCBI as FASTA files of protein sequences. We also analyzed 12 Freshwater microbial genomes mentioned in (Hopkinson and Morel, 2009) making a total of 164 genomes.

### Obtaining the metagenome sequences

The GOS metagenomic sequences and the corresponding metadata were downloaded from the CAMERA portal (Seshadri et al., 2007). The nucleotide sequences were translated in all six frames and all translations with a length less than 25 amino acids were discarded. Since there was a large variation in the number of sequences in each sample, we used the Daisychopper^2^ strategy to randomly select an equal number of sequences from all the samples. From the 44 GOS samples, we selected 30 samples that were obtained from a 0.1–0.8 μm filter and were classified as “Open Ocean” or “Coastal” (Table S2 in Supplementary Material). The sample GS07, from the Northern Gulf of Maine (43.63°N, 66.84°W), had the least number of sequences (50980). Hence, 50980 sequences were randomly selected from each of the other samples for the hmmsearch. The taxonomic profiles for these samples were downloaded from the MG-RAST server^3^.

### Statistical analysis

The results of the *hmmsearch* program were parsed and tabulated as ***m*** × ***n*** matrix (***m*** genomes or metagenomes in rows × ***n*** genes in columns). This matrix was used for making the non-parametric multidimensional scaling (NMDS) plots using the Primer-E v6 software (Clarke, 2006). Discarding gene columns which did not have any hits in any of the genomes, we obtained a 164 × 85 matrix. For the metagenomic samples, an environmental matrix was also created using the metadata provided in CAMERA for the GOS samples. Apart from the geographical coordinates of the samples, the environmental matrix contained “sample depth,” “water column depth,” “temperature,” and “dFe deposition” as variables. The dissolved iron concentration at the surface for the sample coordinates was obtained from the PELAGOS model simulation (Vichi et al., 2007a,b; data kindly provided by Dr. Marcello Vichi). We used the yearly mean concentration of dissolved iron, averaged over the entire period of simulation, i.e., from 1980 to 2002.

Analysis of Similarities (ANOSIM) test for statistically significant differences between prior groupings of the samples made according to taxonomy or location and the Similarity Percentages (SIMPER) analysis comparing relative abundances of genes in the said prior groupings to identify discriminating genes were carried out using Primer-E. Principal Components Analysis (PCA) of the GOS samples using the environmental matrix (Tables S1 and S2 in Supplementary Material) was also performed using Primer-E. Following is a brief description of the non-parametric statistical methods implemented in Primer-E (Clarke, 1993) that we have used here.

#### Data transformations

Whereas the abundance matrices were log-transformed, for the environmental matrix, the variables were individually transformed to reduce the collinearity as much as possible. So, Latitude and Longitude were square-root transformed, “Water column depth” was log-transformed and “dFe deposition” was exponential-transformed.

#### Bray–Curtis similarity

The first step in the analysis of multivariate data was the calculation of a similarity measure between the samples. The similarities between all pairs of samples (the similarity matrix) were then used for a number of analyses. The Bray–Curtis similarity coefficient is the most common measure for comparing ecological samples with species abundance data. The Bray–Curtis measure is independent of scale of measurements (counts, biomass etc.) and joint absences of variables in a pair of samples have no effect on the similarity between them. For two samples *j* and *k* the Bray–Curtis similarity is described by

(1) $$S_{jk}=100\frac{\sum_{i=1}^{p}2\min\left(y_{ij},y_{ik}\right)}{\sum_{i=1}^{p}\left(y_{ij}+y_{ik}\right)}$$

where *y_ij_* and *y_ik_* are the abundance of the *i*th variable in the *j*th sample and *k*th sample respectively and *p* is the total number of variables. The Bray–Curtis dissimilarity is then simply represented as 100 − *S_jk_*.

#### Non-metric multidimensional scaling

Ordination plots visually display the similarity between ecological samples by mapping the high-dimensional community structure to two or three dimensions such that the physical distance between samples on the plot reflects the similarity between their communities.

In an NMDS ordination plot, the distances between the samples (in this case genomes or metagenomes) are first calculated using complete profiles of occurrence of the variables (in our case the iron metabolism genes). The sample objects are then placed randomly in a 2-d space and the Euclidean or physical distance between the objects in 2-d is calculated. This distance matrix is then non-parametrically regressed on to the original distance matrix to calculate a stress value (goodness-of-fit of the regression) that gives an indication of the best fit between the two matrices. The samples are then iteratively rearranged such that the stress value is minimized. The NMDS plot thus is a 2-d representation of the distances between the samples in a high-dimensional space. The distance between two genomes in such an ordination diagram gives an indication of the similarity of their gene profiles. A stress value less than 0.2 combined with an overlay of pre-defined group names provides reliable inferences about the clustering of the samples.

#### Analysis of similarities

The ANOSIM test is the non-parametric multivariate analog of the Analysis of Variance tests for univariate, normally distributed data. Instead of the group means as in the univariate case, here only the rank similarities between the samples in the underlying similarity matrix are considered. For *n* samples having replicates for two or more categories (in our case the taxonomic or ecological niche groups) a test statistic *R* is calculated as follows

(2) $$R=\frac{\bar{r_{B}}-\bar{r_{W}}}{\frac{1}{2}M}$$

where $\bar{r_{W}}$ is average of rank similarities in the replicates within a category, $\bar{r_{B}}$ is the average of rank similarities among all pairs of replicates between the categories, *M* = *n*(*n* − 1)/2 and *n* is the total number of samples. The statistical significance of the observed *R* value is evaluated using the null hypothesis *H*_0_ that there are no differences between the groups of samples. This is accomplished by a permutation test where all the group labels are sequentially applied to all the samples and the *R* statistic recalculated for each permutation. The null hypothesis *H*_0_ is rejected if the observed *R* value lies outside of the distribution of *R* values from the permutation test. For instance if ***t*** *R* values of the *T* total permutations are greater than or equal to the observed *R* value then we can reject *H*_0_ at a significance level of (*t* + 1)/(*T* + 1). This is what is referred to as the Global *R* test, i.e., between all the groupings of the samples. Pairs of the groups were also similarly compared to each other in terms of the *R* statistic and its significance value. Following convention, we tolerated a significance value of up to 5% (Type I error, i.e., rejecting the null hypothesis when it is true) as being small enough to rule out the possibility of *H*_0_ being true.

#### Similarity percentages

This method disaggregates the Bray–Curtis similarity matrix in order to identify the species that contribute most to the differences (average dissimilarities) between the prior groupings of the samples. For two samples *j* and *k* SIMPER calculates the contribution for the *i*th species as follows:

(3) δjk(i)=100|yij−yik|∑i=1p(yij+yik)

The terms *y_ij_* and *y_ik_* are defined as before for Eq. (1). The average contribution $\bar{\delta_{i}}$ of the *i*th species to the overall dissimilarity $\bar{\delta }$ is just the average δ*_jk_*(*i*) for all pairs (*j*,*k*) such that *j* is from the first group and *k* is from the second group. If $\bar{\delta_{i}}$ is high and the standard deviation SD (δ*_i_*) of the δ*_jk_* (*i*) values is low, it implies that this species *i* has a significant contribution to the overall dissimilarity in a majority of pairwise comparisons between the two groups. A high $\bar{\delta_{i}}∕\text{SD}\left(\delta_{i}\right)$ ratio therefore means that species *i* is a good discriminator.

#### Principal components analysis

Principal components analysis is an ordination where the high-dimensional data is represented in terms of two or three orthogonal axes (Principal Components). The procedure involves finding a linear combination of the original variables (first PC) such that the variance of the sample points projected perpendicularly on this new axis is maximized. The second PC is restricted to be perpendicular to the first PC and again chosen in the direction that maximizes the variance of the sample points and so on. The percentage of the variance explained by the first three PCs gives an idea about the loss of information resulting from reducing the dimensions. The variable vectors can be plotted on top of the PCA ordination to visualize the directions of the variable gradients.

### Phylogenetic and positive selection analysis

The 16S rRNA gene sequences for the genomes were retrieved from GenBank along with the *E. coli* 16S rRNA sequence. These were aligned using MUSCLE and imported into the ARB software (Ludwig et al., 2004). A Maximum Likelihood tree was calculated with the FastDNAML (Olsen et al., 1994) implementation in ARB using a filter for base 800 to base 1300 encompassing and extending on both sides, the v6 hypervariable region in *E. coli*. An in-house script was used to calculate the average phylogenetic distance of a gene as follows:

(4) $$P=\text{Avg}D_{p}\left(g\right)$$

where *D_p_*(*g*) is the set of pairwise phylogenetic distances between all pairs of genomes where gene *g* occurs.

For the genes discriminating between taxa or locations, the nucleotide sequences were retrieved from GenBank and the Maximum Likelihood estimations of average pairwise non-synonymous by synonymous mutation (dN/dS) ratios were calculated using CodeML (runmode = −2) from the PAML package (Yang, 2007).

## Results

**Figure 1:**
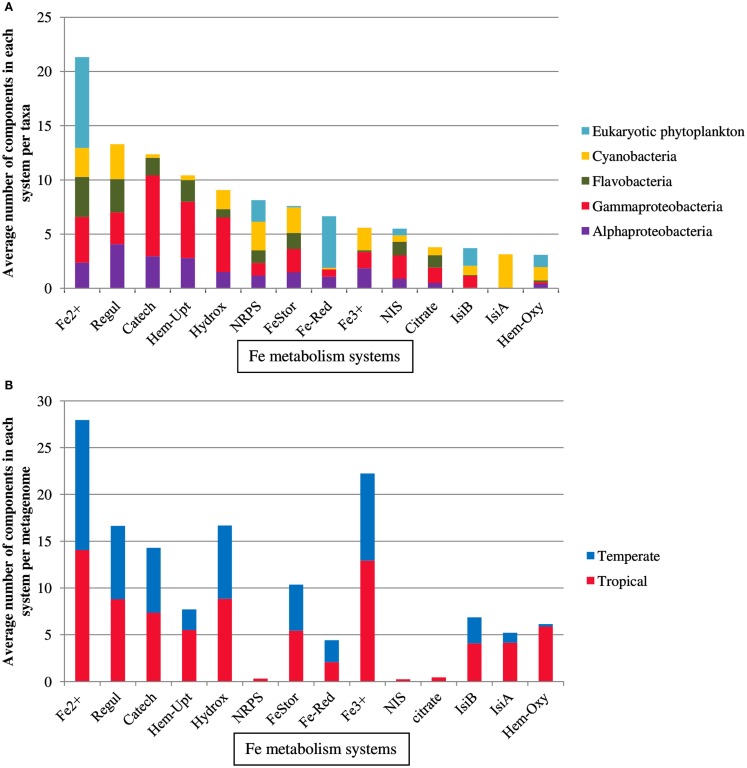
**Distribution of components of Fe-metabolism systems in (A) marine microbial taxa and (B) Temperature groupings of metagenomes**. For detailed description of the components of each system see Table 1.

A set of proteins involved in iron metabolism (Table 1) was recovered from 164 marine microbial genomes belonging to *Cyanobacteria*, eukaryotic phytoplankton, *Alphaproteobacteria*, *Gammaproteobacteria*, and *Flavobacteria* (Figure 1A) using HMMs with optimized thresholds and modified emission probabilities as described earlier (Srivastava et al., 2007). The heme biosynthesis system, the TonB/ExbB/ExbD system, ferredoxin, and the iron-sulfur cluster assembly protein Isca1 (Table 1) were present in almost all prokaryotic genomes and therefore removed from further analysis. The observed abundances of TBD Fe-siderophore uptake systems, components of Fe^2+^ or divalent cation uptake and Fe^3+^ transporters were in agreement with previous reports (Hopkinson and Barbeau, 2012). The TBD uptake systems for catecholate, hydroxamate, and citrate siderophores were more widespread in *Gammaproteobacteria* (60, 55, and 37% of the genomes, respectively) as compared to *Alphaproteobacteria* (24, 16, and 13%, respectively), *Flavobacteria* (13, 8, and 30%, respectively), and *Cyanobacteria* (2, 19, and 19%, respectively). Fe^2+^ or divalent cation transporters were abundant in all the taxa but were most abundant in the eukaryotic phytoplankton genomes (39%). Ferric reductase was characteristic of the eukaryotic phytoplankton group (71%), but was also present in *Cyanobacteria* (2%), *Alphaproteobacteria* (16%), and *Gammaproteobacteria* (10%). Fe^3+^ transporters occurred in *Cyanobacteria* (37%), *Alphaproteobacteria* (33%), and *Gammaproteobacteria* (27%), but were uncommon in *Flavobacteria* (2%) and absent from eukaryotic phytoplankton. NRPS and NIS components involved in siderophore biosynthesis were present in *Alphaproteobacteria* (14 and 16%), *Gammaproteobacteria* (14 and 39%), *Flavobacteria* (14 and 22%), *Cyanobacteria* (32 and 11%), and also in eukaryotic phytoplankton (24 and 11% respectively).

### Siderophore biosynthesis components in phototrophic genomes

**Figure 2:**
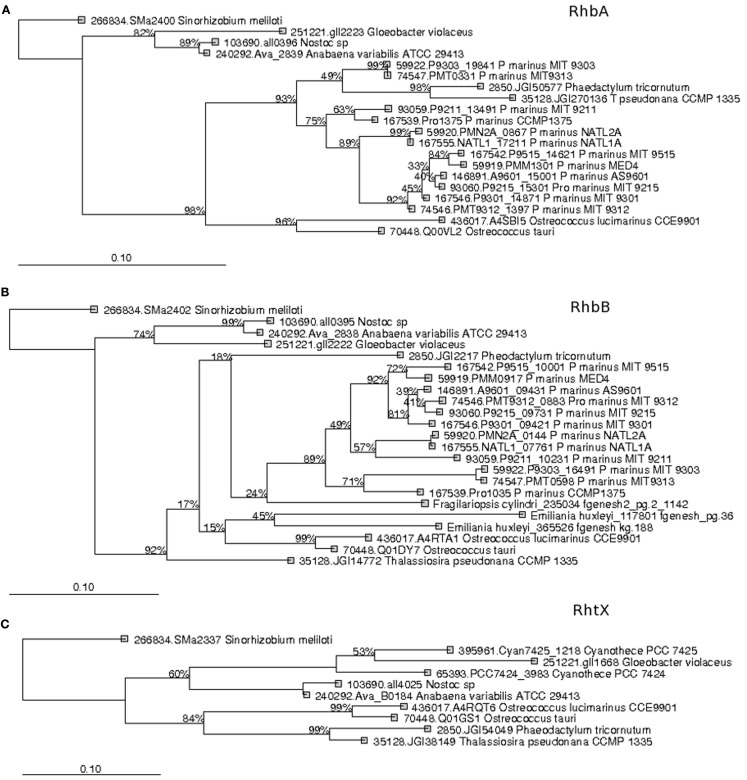
**Phylogenetic trees of (A) RhbA, (B) RhbB, and (C) RhtX protein sequences from Open Ocean picocyanobacteria and eukaryotic phytoplankton recovered in our study**. The tree was constructed using a Neighbor-Joining method, using the Jukes–Cantor correction and a bootstrap test was conducted with 1000 replicates. The scale bar represents 100% estimated sequence divergence.

Recent surveys involving searches of NIS components represented by PFAM domains AlcB (Acetyl transferase) and IucA_IucC (siderophore synthetase for Aerobactin) suggest that none of the eukaryotic phytoplankton and only around 4% of marine picocyanobacteria possess this system (Hopkinson and Morel, 2009; Hopkinson and Barbeau, 2012). Here our HMM search utilized a more extensive set of NIS proteins involved in the biosynthesis of aerobactin, desferrioxamine, and rhizobactin 1021 siderophores (Challis, 2005; Table 1). NRPS was detected in picocyanobacteria *P. marinus* MIT9303 and NRPS along with the NIS component RhbB (a PLP dependent decarboxylase) were detected in *P. marinus* MIT 9303 and MIT 9313. It is possible that the high specificity of our HMM-ModE models led to a slight drop in sensitivity. To confirm whether the other components of this pathway were indeed present in the phototrophic genomes and were being missed due to this lowered sensitivity of HMM-ModE, we used the Search Tool for Interacting Genes/Proteins (STRING) database (Szklarczyk et al., 2011). For a given query sequence, this database identifies a set of proteins that repeatedly co-occur with the query in the genomes of many different organisms. In addition to *P. marinus* MIT9303 and MIT9313, using the *S. meliloti* RhbB sequence as the query, the STRING database showed the co-occurrence of RhbB and RhbA (diaminobutyrate aminotransferase involved in rhizobactin biosynthesis) in *P. marinus* CCMP1375, NATL1A, CCMP1986, MIT9211, MIT9515, MIT9215, MIT9312, NATL2A, AS9601, and MIT9301. A corresponding siderophore uptake gene was not detected in the *Prochlorococcus* genomes. Our profiles detected a putative gene for NRPS in eukaryotic phytoplankton *A. anophagefferens*, *E. huxleyi*, *O. tauri*, *P. tricornutum*, and *T. pseudonana*, and the NIS component RhbB in *E. huxleyi*, *F. cylindrus*, and *O. lucimarinus*. Using the STRING database we detected genes similar to rhizobactin biosynthesis components RhbA and RhbB in *O. tauri*, *O. lucimarinus*, *T. pseudonana*, and *P. tricornutum* along with RhtX, a special permease involved in the uptake of rhizobactin 1021. The sequences identified as RhbA, RhbB, and RhtX from these genomes shared 48.18, 35.48, and 30% identity at the protein level within each group, respectively. A neighbor-joining tree calculated from the multiple sequence alignments of these sequences showed a higher similarity of the Freshwater organisms with the *Sinorhizobium* genes while the eukaryotic sequences clustered with the *Prochlorococcus* sequences (Figure 2). We also detected the NRPS as well as NIS components in the metagenomes, though their abundances were low (Figure 1B).

### Analysis of genomes in terms of their taxonomic affiliation

We generated occurrence matrices showing the abundance of Fe-metabolism proteins (variables) in each genome or metagenome (samples) and used NMDS plots to visualize the clustering of the samples based on similarities of their gene occurrence profiles. The distance between the sample points on a NMDS plot is indicative of the extent to which samples share species (or proteins in this case). We grouped the individual genomes according to either their taxonomic class or to their ecological niches and applied a multivariate non-parametric test (ANOSIM) to check for differences in distribution of the Fe uptake systems across these groupings. For groups that showed a significant difference (a positive ANOSIM *R* value with significance <5%) in the type and frequencies of occurrence of Fe uptake systems, the SIMPER method was used to identify the proteins that contributed the most to this difference (see Materials and Methods for details). The taxonomic groups took into account the heterotrophic genomes, comprised of *Alphaproteobacteria*, *Gammaproteobacteria*, and *Flavobacteria*, and the phototrophic genomes consisting of picocyanobacteria (*Synechococcus* and *Prochlorococcus*), other-*Cyanobacteria* (*Cyanobacteria* excluding picocyanobacteria), and the eukaryotic phytoplankton. The genomes were grouped into niches based on only the isolation location of the source organism, e.g., Open Ocean, Coastal, or Freshwater (Table S1 in Supplementary Material).

**Figure 3:**
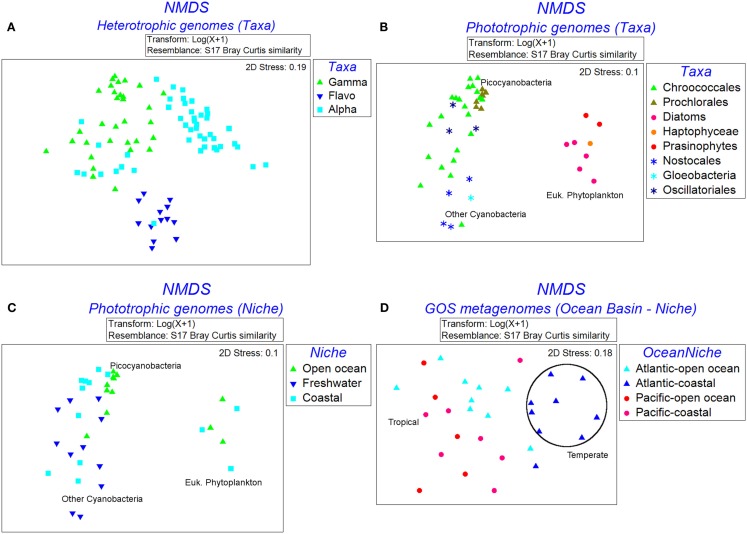
**Non-parametric multidimensional scaling plots showing the clustering of genomes and metagenomes based on the similarities of occurrence patterns of Fe uptake systems**. **(A)** Heterotrophic genomes labeled by taxa, **(B)** phototrophic genomes labeled by taxa, **(C)** phototrophic genomes labeled by niche, and **(D)** GOS metagenomes labeled by marine niche groups.

The NMDS plot of 110 heterotrophic genomes showed three distinct clusters corresponding to the three taxa (Figure 3A). The differences in Fe-metabolism systems among the three groups were statistically significant (Table 2). The greatest diversity of TBD hydroxamate/catecholate siderophore and heme uptake components, and occurrence frequency of bacterioferritin and NIS biosynthesis component RhbB, as identified by SIMPER, was seen in *Gammaproteobacteria* (Table 3). The *Alphaproteobacteria* genomes had the highest occurrence frequencies of Ferric reductase, the Zinc uptake protein ZupT (free Fe^2+^and other divalent cations), and FbpA (Fe^3+^ transporter component) as well as regulatory elements Fur and RirA. The ferric citrate uptake protein FecA, the FeoAB proteins (Fe^2+^ uptake), and Ferritin were amongst the most abundant in *Flavobacteria* and infrequent in the other two groups of heterotrophic bacteria. The regulatory element DtxR was only present in *Flavobacteria* and absent in *Alphaproteobacteria* and *Gammaproteobacteria*.

**Table 2:** **Statistical comparison (ANOSIM) of Fe-metabolism systems in various groupings of genomes and metagenomes**.

Groups	*R* statistic	Significance level %
**GENOME CATEGORIES**		
**Heterotrophs-taxa**		
Gammaproteobacteria, Flavobacteria	0.862	0.1
Gammaproteobacteria, Alphaproteobacteria	0.455	0.1
Flavobacteria, Alphaproteobacteria	0.657	0.1
**Phototrophs-taxa**		
Picocyanobacteria, other-Cyanobacteria	0.406	0.1
Picocyanobacteria, Eukaryotic phytoplankton	0.942	0.1
Other-Cyanobacteria, Eukaryotic phytoplankton	0.964	0.1
**Phototrophs-niche**		
Open Ocean, fresh water	0.332	0.1
Open Ocean, coastal	0.125	3.1
Fresh water, coastal*	0.074	8.8
**GOS Groups**		
North Atlantic Open Ocean, South Pacific	0.142	3.7
Tropical, temperate	0.469	0.1
Coastal, Open Ocean	0.201	0.4

An *R* value with significance level more than 5% implies that the corresponding groups cannot be statistically differentiated from each other. The insignificant pair of groupings is marked with *.

**Table 3:** **Fe-metabolism protein components discriminating between the three major taxonomic groups of heterotrophs (A) and phototrophs (B) and groupings of the GOS metagenomes (C) identified using the Similarity Percentages (SIMPER) method**.

Protein	(A) Heterotrophs			(B) Phototrophs			(C) GOS metagenomes					
	Gamma	Alpha	Flavo	Picocyano bacteria	Other Cyano bacteria	Eukaryotic Phyto plankton	Fe conc groups		Temperature groups		Niche groups	
							Atlantic Open Ocean	Pacific Ocean	Tropical	Temperate	Open Ocean	Coastal
**HEME TBDT UPTAKE**												
HasF	**0.73**	0.09										
PhuR	**0.45**	0.19	0.43									
HmuT	**0.43**	0.28	0.1				0.08	**0.96**	**0.56**	0	0.28	**0.52**
HmuU	**0.81**	0.67	0.25	0.13	**0.48**		0.15	**0.83**	**0.55**	0.31	0.43	**0.53**
HmuV	**0.48**	0.33	0.1				0.2	**0.35**	0.27	**0.53**	0.28	**0.4**
HemU	0.23	0.1	**0.45**			0						
**HYDROXAMATE SIDEROPHORE UPTAKE**												
FhuA	**0.75**	0.41	0.36	0.2	**0.93**	0						
IutA	**0.46**		0.05			0						
RhtX				0.13	**0.43**	0						
**CATECHOLATE SIDEROPHORE UPTAKE**												
BtuF	**0.4**	0.06										
BtuB	**0.91**	0.33	0.05									
**CITRATE SIDEROPHORE UPTAKE**												
FecA	0.38	0.07	**0.52**									
**Fe^3+^ TRANSPORTERS**												
IdiA				**0.79**	0.31	0	**1.79**	1.66	**1.72**	1.43	**1.82**	1.49
HitB	**0.55**	0.53	0.05	**0.69**	0.54	0	**1.03**	0.73	**0.82**	0	**0.92**	0.32
FbpA	0.43	**0.59**	0.05				0.46	**0.61**	**0.52**	0.26	**0.54**	0.37
**FREE Fe^2+^ UPTAKE**												
FeoA	0.45	0.09	**0.69**	0.31	**0.61**	0	**0.63**	0.36	0.51	**1.38**	0.89	**1.29**
FeoB	0.48	0.12	**0.69**	0.3	**0.63**	0	**0.99**	0.9	0.9	**1.67**	0.48	**0.97**
ZupT	0.27	**0.49**	0.38			**1.52**	0.12	**1.29**	1.19	**1.25**	1.15	1.25
FTR1				0.17	**0.39**							
MgtE						**0.4**	3.33	3.2				
**REGULATORY ELEMENTS**												
Fur	1.11	**1.36**	1.03			0			**3.72**	3.34	3.71	3.54
RirA	0.55	**0.63**										
DtxR	0	0	**0.81**				0.95	**1.4**	**1.22**	0.96	1.13	**1.16**
**STORAGE**												
BfrB	**0.49**	0.15										
Ferritin	0.67	0.49	**0.87**	1.02	**1.56**	0.09	**1.82**	1.41	1.51	**1.69**	**1.6**	1.53
**NRPS INDEPENDENT SIDEROPHORE SYNTHESIS**												
RhbB	**0.62**	0.42	0.52									
NRPS	0.46	**0.59**	0.49	0.36	**1.67**	0.95						
Ferric reductase	0.44	**0.65**				**1.48**	**1.29**	1.27	1.34	**2**	1.2	**1.79**
IsiA				1.27	1.27	0	**1.72**	1.34	**1.49**	0.17	**1.82**	0.55
IsiB	**0.62**	0.02		0.54	0.6	**0.87**	1.28	**1.35**	**1.3**	0.36	**1.37**	0.76

*The numbers are average log abundances of components that were discriminating between groups with the highest abundance in a given category (A, B, or C) given in bold. An empty cell means that the component might be present in all the groups but is not discriminating between groups*.

Figure 3B shows the NMDS plots labeled by taxonomy for the 54 phototrophic genomes. The *Cyanobacteria* and eukaryotic genomes formed separate clusters. Within the cyanobacterial cluster the picocyanobacteria, which had the highest number of representative genomes, and other-*Cyanobacteria* formed sub-clusters. The phototrophic genomes showed a statistically significant difference between the eukaryotic phytoplankton, picocyanobacteria, and other-*Cyanobacteria* groups (Table 2). The Fe uptake components which showed a marked difference in abundances across the phototrophic genome groups are given in Table 3. The picocyanobacteria were characterized by the periplasmic Fe^+3^ transport components IdiA and HitB which were infrequent in the other-*Cyanobacteria* and not detected at all in the available eukaryotic phytoplankton genomes (Table 3). On the other hand, GTP driven Fe^2+^ uptake components FeoAB, FTR1, and hydroxamate uptake components FhuA and RhtX were largely absent from picocyanobacteria and eukaryotic phytoplankton but were present in the other-*Cyanobacteria* group. Similarly, high occurrence frequencies of the Zinc transporter ZupT, Ferric reductase, IsiB, and NRPS were characteristic of the eukaryotic phytoplankton as compared to the *Cyanobacteria*.

### Analysis of genomes in terms of their ecological niches

The Open Ocean niche group of the phototrophic genomes (Figure 3C) was significantly different from the Coastal and Freshwater groups (Table 2). We observed that the TBD siderophore/heme uptake components as well as the Feo system for Fe^2+^ uptake were most widespread in the Freshwater niche, relatively less common in Coastal niche and rarely represented in the Open Ocean niche (Table 4). FTR1 (direct Fe^2+^ uptake), ferritin, HO1 (heme oxygenase), and NRPS were abundant in Freshwater niche. The Fe^3+^ transporter components (IdiA and HitB) were evenly present in all the three niches. The NIS biosynthesis protein RhbB was most abundant in the Coastal niche. The Open Ocean niche had a greater abundance of IsiA and IsiB than both other niches. The niche groups in the heterotrophic genomes were not distinguishable from each other in terms of the frequencies of iron uptake genes (Table 2).

**Table 4:** **Proteins discriminating between aquatic niche groups in phototrophic genomes identified using the Similarity Percentages (SIMPER) method**.

	Phototroph genomes (niche)		
Protein	Open Ocean	Coastal	Freshwater
**HEME TBDT UPTAKE**			
HasF			
PhuR			
HmuT			
HmuU	0.12	0.19	**0.36**
HmuV			
HemU			
**HYDROXAMATE SIDEROPHORE UPTAKE**			
FhuA	0.12	0.24	**0.87**
IutA	0.04		**0.32**
FhuB	0		**0.26**
FhuC	0.06		**0.23**
RhtX	0.06	0.21	**0.38**
**CITRATE SIDEROPHORE UPTAKE**			
FecB	0.13	0.31	**0.41**
**Fe^3+^ TRANSPORTERS**			
IdiA	0.53	0.63	**0.68**
HitB	0.57	**0.59**	0.58
FbpA			
**Fe^2+^ UPTAKE**			
FeoA	0.19	0.3	**0.74**
FeoB	0.17	0.38	**0.65**
ZupT	**0.28**	**0.28**	0.17
FTR1	0.04	0.03	**0.58**
MgtE	0.64	0.67	**0.71**
**REGULATORY ELEMENTS**			
Fur	1.09	1.27	**1.51**
RirA			
DtxR			
**STORAGE**			
BfrB			
Ferritin	0.76	1.13	**1.41**
**NRPS INDEPENDENT SIDEROPHORE SYNTHESIS**			
RhbB	0.12	**0.43**	0.32
NRPS	0.49	0.67	**1.3**
Fe-Red	**0.35**	0.16	0.15
IsiA	**1.51**	0.89	1.05
IsiB	**0.79**	0.35	0.68

*The numbers are average log abundances of components that were discriminating between groups. The abundances for the categories having the highest values for a given component are displayed in bold. An empty cell means that the component might be present in all the groups but is not discriminating between groups*.

### Environmental characterization of the GOS metagenomes

**Figure 4:**
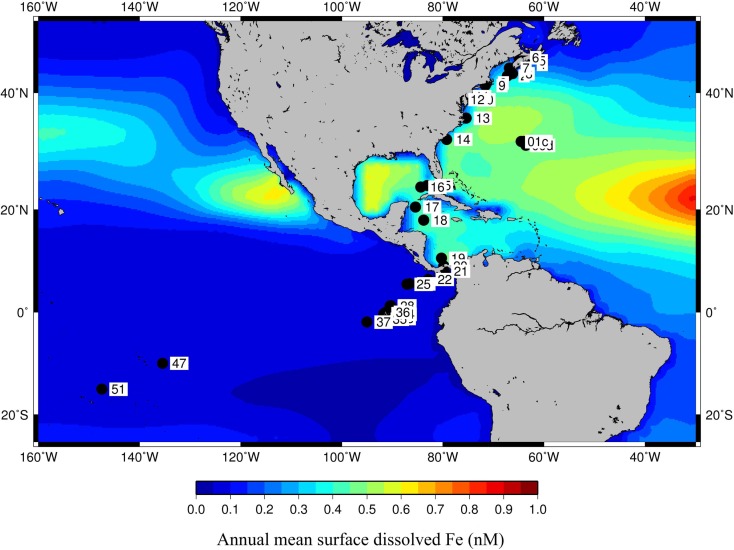
**Coordinates of GOS metagenome samples overlayed on dissolved Fe concentrations as predicted by PELAGOS model**.

Figure 4 shows the GOS sample locations overlayed on the annual mean surface bioavailable dFe from the PELAGOS model (Vichi et al., 2007a,b). This model couples the biogeochemical fluxes with global ocean general circulation models. The iron dynamics in PELAGOS include fluxes for uptake of bioavailable Fe by phytoplankton, loss by turnover/cell lysis, and predation. The only external function that forces these fluxes is the monthly deposition of atmospheric Fe and a dissolution fraction of the Fe dust which is set at 1%. We used the surface dFe concentration predictions from the PELAGOS model to characterize the aquatic niches. Since we were using model-derived dFe concentrations in place of actual observations, we compared the dFe concentrations used in this study with those used in Toulza et al. (2012). For the 22 GOS samples where dFe concentration predictions from both of these models were available, an *R*^2^ of 0.849 in a linear regression of the dFe values showed a good correlation between the outputs of the two models (Table S2 in Supplementary Material). There was a marked difference in the surface dFe concentration in samples collected from the Atlantic and the Pacific basins. However, apart from the dFe differences in the niches (Open Ocean or Coastal) defined in Toulza et al. (2012), the spatial distribution of the samples across a wide range of latitudes also resulted in a separation along a temperature gradient (Temperate vs. Tropical) (Rusch et al., 2007).

**Figure 5:**
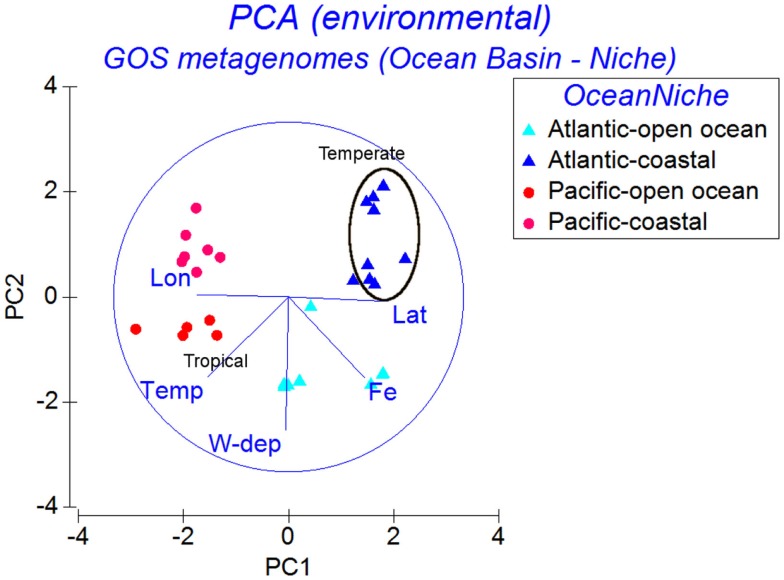
**Principal Components Analysis of GOS samples using similarities of the environmental parameter profiles**. The samples are labeled according to the ocean basins (Atlantic or Pacific) combined with niche groups (Open Ocean or Coastal).

The environmental matrix for the 30 GOS samples belonging to the Open Ocean and Coastal niche groups was subjected to a PCA (Figure 5). The Atlantic and Pacific samples were separated along the first PC, where the environmental parameters accounting for the separation were Longitude, Latitude, and Temperature. Within each ocean basin group (Atlantic or Pacific), along the second PC, the water column depth gradient separated the samples into Open Ocean and Coastal groups. The dFe gradient separated the Atlantic Open Ocean samples from the Pacific Open Ocean and Coastal samples (Figure 5). The first PC, which included the temperature gradient in addition to the dFe, also separated the samples into Temperate and Tropical groups (Figure 5).

**Figure 6:**
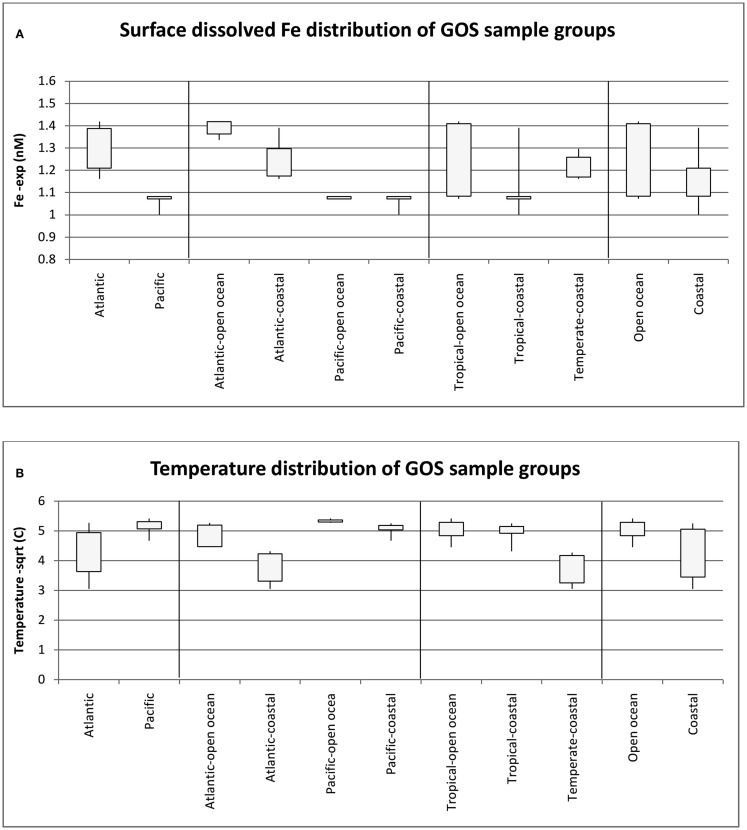
**Median and range of dissolved Fe concentrations (A) and Temperature (B) for the GOS metagenome sample groups used in the study**. Numbers of samples in the groups are as follows: Atlantic (18), Pacific (12), Atlantic Open Ocean (9), Pacific Open Ocean (5), Pacific-Coastal (7), Tropical-Open Ocean (11), Tropical-Coastal (11), Temperate-Coastal (8), Open Ocean (14), Coastal (16).

To further understand the interaction between dFe and temperature and to define meaningful niche groups with contrasting parameters, we compared the median and range of the dFe concentrations as well as the temperatures (suitably transformed as described in the methods) for different group of GOS samples (Figures 6A,B respectively). There was a significant difference in dFe concentrations in the Pacific and Atlantic samples (single factor ANOVA, *p* value = 6.31E−09), and their median temperature difference was also significantly different (*p* value < 0.0005). The most noticeable difference of Fe concentrations was seen in the Atlantic Open Ocean samples and Pacific (Open Ocean and Coastal) samples (*p* value 1.3E−11) whereas the corresponding temperature difference between these groups was not significant (*p* value 0.27 at α = 0.01). Conversely, the Temperate, Coastal samples were primarily separated from the Tropical (Open Ocean and Coastal) samples by a temperature difference (*p* value 2.1E−07) with no difference in the corresponding average Fe concentrations (*p* value 0.3 at α = 0.01). Consequently, only these three pairs of groups (Atlantic Open Ocean vs. Pacific, Temperate vs. Tropical, and Open Ocean vs. Coastal) were further analyzed for the differences in their taxonomic profiles and their associated Fe-metabolism systems.

### Taxonomic distribution and Fe-metabolism components represented in the GOS metagenome groups

The taxonomic profiles (the occurrence of sequences from various taxonomic groups) of the aforementioned GOS metagenome groups were obtained from MG-RAST (Meyer et al., 2008) using an *E*-value cut-off of 1E−15, minimum percent identity 50, and minimum alignment length 100. Using the set of sequences with a clear taxonomic identification we further processed the data to obtain the percentage contribution of each taxonomic group in the metagenomic groupings (Table 5). The Fe-metabolism protein frequency matrix was analyzed for differences in Fe-metabolism components across the GOS metagenomic groupings. The NMDS plots labeled by Ocean Basin – Niche categories as well as the Temperature – Niche groups are shown in Figure 3D. The ANOSIM tests for all these groups were significant (Table 2), suggesting that the distribution of Fe uptake system components was different. However, based on the environmental characterization (see the section above), we selected only the samples from Tropical vs. Temperate and Atlantic Open Ocean vs. Pacific for further analysis with SIMPER along with the previously defined Open Ocean vs. Coastal samples (Toulza et al., 2012). Differences in gene abundances between these groups are given in Table 3. The average abundance of the heme uptake machinery HmuTUV was higher in the Pacific, Tropical, and Coastal groups whereas the Fe^3+^ uptake (IdiA, HitB) and IsiA had a higher representation in the Atlantic Open Ocean (compared to the Pacific), Tropical (compared to Temperate), and Open Ocean (compared to Coastal) groups. The Fe^2+^ uptake components (FeoAB) and Ferric reductase were more abundant in Atlantic Open Ocean, Temperate, and Coastal groups. The Flavodoxin protein IsiB and heme oxygenase HO1 had a higher average abundance in the Pacific, Tropical, and Open Ocean groups. While ZupT (zinc uptake protein) was more abundant in Pacific, Temperate, and Coastal groups, the storage protein ferritin had a higher abundance in Atlantic Open Ocean, Temperate, and Open Ocean groups.

**Table 5:** **Taxonomic profiles of GOS metagenome niche groupings**.

Taxonomic groups	Fe concentration groups		Temperature groups		Niche groups	
	Atlantic Open Ocean	Pacific	Tropical	Temperate	Open Ocean	Coastal
Alphaproteobacteria	41.80	22.97	36.18	16.60	16.57	35.33
Gammaproteobacteria	26.97	24.21	19.99	39.20	35.16	20.18
Flavobacteria	6.26	22.68	6.22	12.47	2.64	7.51
Picocyanobacteria	13.61	1.01	11.55	0.28	5.37	8.86
Other-Cyanobacteria	0.20	0.55	0.23	0.08	0.53	0.11
Eukaryotic phytoplankton	0.05	0.94	0.04	0.49	0.04	0.16

*The numbers indicate the percentage dominance of the taxa in the metagenome groups*.

### Phylogenetic spread and non-synonymous mutation rate of selected genes

**Figure 7:**
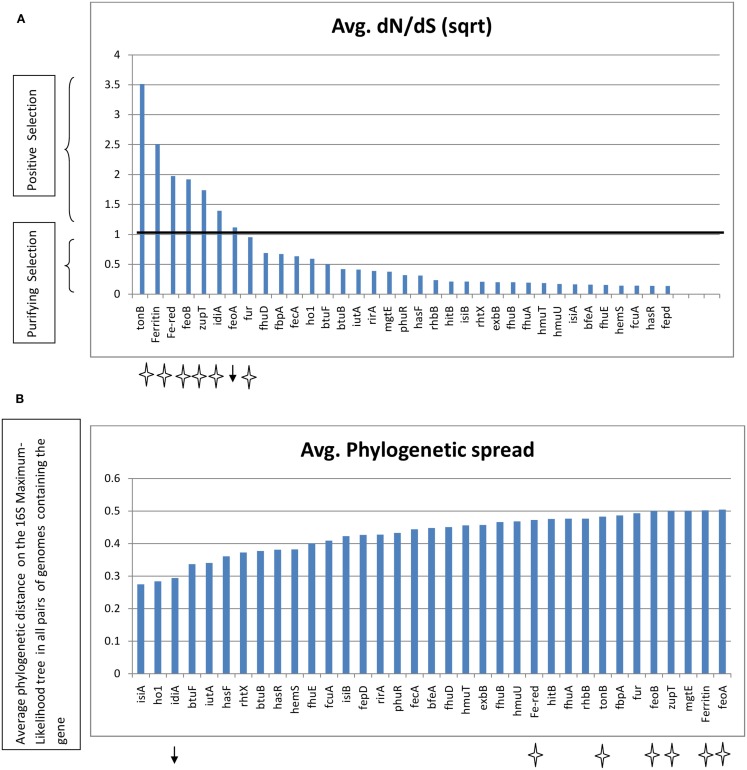
**(A)** Average dN/dS ratios of selected genes. The genes are sorted by highest dN/dS value. **(B)** Phylogenetic spread as defined by maximum phylogenetic distance among the genomes possessing the gene. The black line marks the dN/dS value = 1. Genes with dN/dS >1 and a wide phylogenetic spread are marked with stars. Genes with dN/dS >1 and a narrow phylogenetic spread are marked by an arrow. Fe-red – Ferric reductase.

The nucleotide sequences of some of the abundant genes, extracted from the genomes, were analyzed for the rate of non-synonymous mutations. Because non-synonymous mutations result in amino acid replacement, they are often eliminated by purifying selection, a form of natural selection that selectively removes deleterious mutations. Under certain selection pressures, non-synonymous mutations might be retained when they are advantageous (known as positive selection). The dN/dS ratio therefore provides a measure of the selection pressure operating on a gene. The dN/dS ratio for some of the genes was plotted (Figure 7) along with their average phylogenetic spread (the average phylogenetic distance among the genomes possessing the gene, calculated from a Maximum Likelihood tree of 16S rRNA gene sequences of the genomes). Ferric reductase, *feoA*, *feoB*, *idiA*, and the zinc uptake gene *zupT*, *tonB*, and ferritin genes all had a dN/dS value >1, indicating that non-synonymous mutations were possibly beneficial for these genes and that they were evolving rapidly under positive selection. With the exception of the *idiA* gene, all of these genes also had a wide phylogenetic spread indicating that they were present in a wide range of taxonomic groups (Figure 7). The remaining genes analyzed had a dN/dS ratio <1 (28 out of 35), indicating that they were under purifying selection pressure. All proteins involved in siderophore biosynthesis or high-affinity uptake systems for hydroxamate or catecholate siderophore/heme or vitamin B12 were undergoing purifying selection. The regulatory element *fur*, fbp-family gene *fbpA*, *hitB*, *isiA*, *isiB*, and the Mg^2+^ transporter *mgtE* were also included in the category of purifying selection and, with the exception of *isiA*, retained a wide phylogenetic spread among the marine genomes.

## Discussion

### Multivariate approach using functional gene specific HMMs

Hidden Markov models of protein families or folds have been routinely used in genome wide studies of protein functions such as metal binding capabilities (Dupont et al., 2006, 2010) or Fe transport (Hopkinson and Barbeau, 2012). Some Fe uptake proteins have multiple domains, which could be shared among different functional classes. For example, the TonB-box is a conserved motif which is common to all TBD receptors binding to different substrates (hydroxamate, catecholate, heme, or citrate). The GTP-binding domain of FeoB which is conserved and involved in various other functions could also lead to increased FP, especially in the metagenomic sequences (Hopkinson and Barbeau, 2012). It has been shown earlier that the specificity of the identification of function at the substrate binding level can be increased by modifying the HMM using information from the negative training sequences (i.e., sequences of the same fold or family but having different substrate binding function) (Srivastava et al., 2007; Desai et al., 2011). We constructed profile HMMs of Fe-metabolism proteins covering most known Fe uptake systems (Table 1), using the HMM-ModE protocol (Srivastava et al., 2007) to increase the specificity at the substrate binding level. Our finding that TBD hydroxamate uptake components are relatively abundant in the metagenomes (Figure 1B) was in agreement with the fact that hydroxamate siderophores are abundant in seawater and constitute upto 5% of the dFe concentration in the Atlantic Ocean (Macrellis et al., 2001; Mawji et al., 2008; Velasquez et al., 2011; Gledhill and Buck, 2012).

Our method also provided a wider range of components to search for in the genomes and metagenomes as demonstrated by the hits obtained for the proteins RhbA (diaminobutyrate-2-oxoglutarate aminotransferase) and RhbB (*L*-2,4 diaminobutyrate decarboxylase) that suggest the presence of some of the components for rhizobactin siderophore biosynthesis in *Cyanobacteria* and eukaryotic phytoplankton genomes (Table S1 in Supplementary Material). However, the confirmation of siderophore biosynthesis pathways in these organisms will depend in part on the positive identification of the remaining genes for the many necessary components that could not be detected in these genomes using the HMMs and the STRING database as input for the search. Also, the homologs of RhtX detected in the eukaryotic genomes from the STRING database, were mostly annotated as Acetyl CoA transporters and had only a weak similarity with the *S. meliloti* RhtX protein (Tables S3–S5 in Supplementary Material), making their identification tenuous without supportive evidence. Siderophore biosynthesis has been reported in some *Synechococcus* species (Wilhelm and Trick, 1994; Ito and Butler, 2005; Hopkinson and Morel, 2009) and predicted to be present in the prasinophyte *O. lucimarinus* (Palenik et al., 2007) suggesting that they may be more widespread than originally thought. Recent reports have shown the production of Fe-binding ligands by microbial communities dominated by diatoms under Fe-depleted conditions with a distinct correlation between Fe-binding ligand concentration and diatom growth (Buck et al., 2010; King et al., 2012).

The increased specificity (and the related sensitivity drop) of the HMM search implies that we might miss identifying Fe-metabolism components in the genomes and metagenomes and our bottom-up approach (starting from known sequences) would prevent us from discovering novel or highly diverged forms of these Fe uptake systems. Also, for the metagenomes, since we sampled equal numbers of sequences, the reduced search space led to the under-representation of some Fe-metabolism systems. For example, even though the hydroxamate TBD uptake systems turned up as discriminating between the genome groups, they were not detected in sufficient numbers or discriminating between metagenome groupings (Table 3). To see if this under-representation was a result of the method and the reduced search space, we scanned all the sequences in the metagenomes with our profiles (Figure 1B). We did find components of hydroxamate, catecholate, and citrate siderophore uptake along with heme degrading oxygenases in all the groups of samples. However, since our aim was to compare samples from different locations, it was important to minimize the effect of sequencing effort (Gilbert et al., 2010), and so, all our comparisons were performed with the sampled metagenome sequence data.

Multivariate analysis is increasingly being applied in microbial ecology studies, for example, to trace the seasonal variation in bacterial communities (Gilbert et al., 2010), compare communities from different niches (Dinsdale et al., 2008) or investigate the correlation of environmental factors with the observed community structure and function in the GOS metagenomes (Gianoulis et al., 2009; Raes et al., 2011). The traditional concept of the species as a fundamental unit of biological diversity does not apply to prokaryotes. A new bacterial equivalent of a species (an ecotype) arises when a bacterial lineage starts utilizing a different set of resources for occupying a new ecological niche or microhabitat (Cohan, 2002). The bacterial genome has the capability to re-organize itself according to environmental cues in a given niche by mechanisms such as horizontal gene transfer (Thompson et al., 2011; Hopkinson and Barbeau, 2012) and hence, could be viewed as an assemblage of genes. In our analysis, we therefore used the profiles of abundances of the Fe-metabolism components (as variables or species that are subject to change) in the genomes or metagenomes (the ecological equivalent of samples) to calculate the similarities between pairs of samples, and further, between *a priori* groupings of the samples. Our multivariate analysis detected distinct patterns of co-occurrence in the groups (both genomes and metagenomes) including the co-occurrence of multiple components of the same system. For example, in most of the comparisons of the groups that we performed, all components of the heme uptake machinery HmuTUV, Fe^3+^ transporters, or the FeoAB system were reported as significant. Concomitantly, the characteristic occurrence patterns for each genome group (Figure 1) were also captured accurately. The reliance of picocyanobacteria and *Alphaproteobacteria* on Fe^3+^ transporters and their absence from *Flavobacteria*, or the widespread use of FeoAB based Fe^2+^ uptake by *Flavobacteria* and its absence from picocyanobacteria and *Alphaproteobacteria* are facts borne out by recent studies (Hopkinson and Barbeau, 2012) which were also apparent in our analysis. Thus, the multivariate analysis of uptake systems in genomes, using the genome as a unit of ecological treatment, validated our approach and demonstrated that it could be used to determine similar differences between the niche groups of genomes and metagenomes.

### Environmental enrichment of Fe uptake system components correlates with the distribution of dominant taxonomic groups

The classification of a microbial genome as either Open Ocean or Coastal in our study was based on the location where they were isolated and does not preclude the possibility of it being present in other niches. However, the abundance of a plethora of Fe uptake systems along with both NIS and NRPS siderophore biosynthesis components in Freshwater and Coastal phototrophs potentially reflected the diversity of Fe-binding ligands in these niches as compared to the oligotrophic Open Ocean organisms where only the homeostasis proteins IsiA, IsiB, and Ferric reductase were abundant (Table 3). The GOS metagenomes provided an opportunity for the same comparison at the community level and also afforded a correlation of these differences to temperature, dFe concentration, and a distinction between Open Ocean and Coastal niches which were set apart from each other by water column depth (Figure 5). The SIMPER analysis demonstrated that certain genes were enriched in a particular niche, i.e., they had a distinct environmental signature.

A detailed analysis of the occurrence patterns of major microbial taxa in the same GOS metagenomic groupings (Table 5) was instructive of how the distribution of Fe-metabolism systems was effected by the environmental parameters. The Tropical metagenomes were dominated by SAR11 cluster and other *Alphaproteobacteria* (36.18% of total identified taxa) and picocyanobacteria (11.55%). These taxa were notably under-represented in the Temperate metagenomes (16.6 and 0.28% respectively). On the other hand the Temperate metagenomes were mainly composed of Flavobacteria (12% as compared to 6% in Tropical) and *Gammaproteobacteria* (39%; Table 5). The major differences in the Fe uptake systems between these groups, i.e., more Fe^3+^ transporters, IsiA, and IsiB in Tropical vs. more Fe^2+^ uptake, ferritin and Ferric reductase in Temperate were in accordance with the differences between the picocyanobacteria and *Alphaproteobacteria* on one hand and the *Flavobacteria* on the other (Table 3). The HmuTUV system, though decidedly more widespread in *Gammaproteobacteria*, is nonetheless present in *Alphaproteobacteria* genomes (Table 3). The combined contribution of *Alphaproteobacteria* and *Gammaproteobacteria* sequences is roughly the same in both groups (58.17 and 55.80% of all Tropical and Temperate sequences respectively; Table 5). We also compared just the coastal samples from the Tropical and Temperate zones (Table S2 in Supplementary Material) to remove the effect of Open ocean and Coastal locations and the results were unchanged. The impact of temperature on the taxonomic and functional diversity of the GOS samples is well established (Rusch et al., 2007; Raes et al., 2011). Here, we showed that temperature, potentially, also has an impact on the Fe uptake system distribution.

In the metagenomic groups separated by dFe concentrations, picocyanobacteria were well represented in the Atlantic Open Ocean (Fe-replete) samples whereas *Alphaproteobacteria* clades dominated the Fe-depleted Pacific samples. Again in this case, Fe uptake system distribution between the two groups (Table 3), i.e., more Fe^3+^ transporters, Fe^2+^ uptake, and IsiA in the Atlantic vs. more HmuTUV, FbpA, ZupT, and IsiB in the Pacific, was a reflection of the differences between corresponding dominant taxonomic groups (Table 5).

Niche specific adaptations and diversity in Fe uptake mechanisms among *Cyanobacteria* are well documented. For example, siderophore production and its associated receptor mediated uptake is more common in freshwater and coastal cyanobacteria but is not the preferred iron acquisition strategy in open ocean *Cyanobacteria* (Webb et al., 2001; Palenik et al., 2006; Hopkinson and Morel, 2009). Our results of the niche analysis of phototrophic genomes also showed this distinction (Table 4). Recent sequencing of various *Synechococcus* genomes points to the presence of Fe^2+^ uptake in coastal strains (Palenik et al., 2006) as an adaptation to the higher concentrations of bioavailable Fe^2+^ in coastal regions (Kuma et al., 1992). The upregulation of IdiA as a response to Fe limitation both in culture as well as in the Fe-limited open ocean is well known in *Cyanobacteria* (Rusch et al., 2010; Thompson et al., 2011). Additionally, presence of Fe^3+^ transporters, and lack of TBD Fe uptake systems in Candidatus *Pelagibacter ubique*, an open ocean alphaproteobacterium might also be a niche specific adaptation (Smith et al., 2010). In light of the above facts and because of possible differences in Fe-speciation (Boye et al., 2003; Buck and Bruland, 2007), niche specific adaptive differences in Fe-metabolism gene profiles were expected between the Open Ocean and Coastal groups. However since all the Temperate samples were also Coastal, some of the differences between Temperate and Tropical groups might, in essence, be reflected in the differences between the Coastal and Open Ocean niche groups. For example, the FeoAB system and Ferric reductase (over-represented in Temperate) were also more abundant in the Coastal group. Additionally, the Coastal group also had higher abundance of the HmuTUV. The Open Ocean group on the other hand had higher occurrence of Fe^3+^ transporters, IsiA, and IsiB (Table 3). Taxonomically, the Coastal group was again dominated by *Alphaproteobacteria* and *Gammaproteobacteria* together constituting around 55% of all identifiable sequences in the group. The Open Ocean group also had a similar proportion of the proteobacteria (roughly 52% of total). The *Flavobacteria* and picocyanobacteria were both over-represented in the Coastal group (Table 5). But within the picocyanobacteria the pelagic *Prochlorococcus* was more abundant in Open Ocean while the Chroococcales (*Synechococcus* spp., *Synechocystis* spp. etc) were almost six times more abundant in the Coastal group than in Open Ocean (data not shown). The over-representation of Fe^3+^transporters, IsiA, and IsiB in the Open Ocean could be explained by the abundance of *Prochlorococcus* while the FeoAB and Ferric reductase in the Coastal group could be due to the abundant Flavobacteria. In conclusion for each metagenomic group the Fe-metabolism component was representative of the taxonomic groups dominant in those metagenomic groups. The environmental niche defined the taxonomic dominance which in turn led to the enrichment of particular Fe uptake components in that particular niche.

### Differential selection pressures on Fe responsive genes

The genes for some of the proteins which were discriminatory between the niches also showed evidence of positive selection pressure at the sequence level (Figure 7). Most of these fast evolving genes perform generalized functions, which could involve interacting with multiple ligands. Both *idiA* and *fbpA* bind Fe^3+^, but only *idiA* was under positive selection pressure while *fbpA* was under purifying selection pressure. However the phylogenetic spread of FbpA was high and occurrence of *idiA* was more or less confined to the *Cyanobacteria*. It has been postulated that Fe^3+^ transporters such as *idiA* interact with Fe bound organic complexes rather than with free Fe^3+^(Hopkinson and Barbeau, 2012). Hydroxamate siderophores have been detected throughout the Atlantic Ocean (Mawji et al., 2008), and it could be believed that by virtue of broadly specific Fe^3+^ transporter *idiA* or *fbpA* the Ópen Ocean cyanobacteria could make a variety of siderophores available to themselves. Similarly the non-specific nature of Ferric reductase mechanism (Schroder et al., 2003) might provide an edge to organisms by catering Fe bound to diverse ligands in an organically rich environment, as evident by our finding that Ferric reductase mechanism was relatively abundant in North Atlantic Open Ocean and Temperate (Coastal) groups of metagenomes. An exception is the abundance of TBD mechanisms in South Pacific group which is known to be oligotrophic. The components of the specialized TBD siderophore/heme uptake systems or the enzymes involved in siderophore biosynthesis, heme oxygenase etc were undergoing purifying selection and the phylogenetic spread was also low for many of them. This suggested that both, the design of the siderophore and the uptake machinery employed are species specific and not much variation at the amino acid sequence level is allowed in the proteins involved. In addition, because of the cost involved in the production of siderophore and the uptake machinery, when present they should confer a definite competitive advantage to the organism. The open ocean environment is highly diffusive and an organism in such a niche cannot benefit from the possession of Fe uptake systems specific for a particular Fe-binding ligand or production of siderophores (Hopkinson and Morel, 2009). This could explain our observation that the specialized TBD uptake systems (e.g., HmuTUV) were more represented in the nutrient rich Coastal niche than in the Open Ocean. These dN/dS calculations were performed using only the genomic sequences. Thus there is a possibility that the results obtained were biased by the high specificity of the HMMs and that the actual rates of evolution in environmental sequences may differ. However, the HMM-ModE protocol has a sensitivity of ∼90 and ∼96% specificity for annotating complete gene sequences in bacterial genomes (Desai et al., 2011). A recent study of evolutionary rates of genes from environmental populations of coastal *Synechococcus* spp. reported that around 98% of the genes evolved under purifying selection (Tai et al., 2011). Also, the relationship between selection pressure and dN/dS ratio is only valid over long evolutionary time scales when comparing the sequences of divergent species (Kryazhimskiy and Plotkin, 2008).

In this time of rapid change in global oceanic conditions, the selection mechanisms operating on the evolution of genes conferring adaptation to a particular oceanic habitat are continuously shaping the genetic composition of microbial communities. We defined aquatic ecological niches for the GOS metagenomes in terms of dFe concentrations and temperature and investigated the differences in distribution of the taxonomic groups as well as the Fe-metabolism systems between these niches. The distribution of the Fe uptake proteins correlated with the taxonomic distribution of the organisms that possessed these systems, suggesting a role for temperature and Fe in shaping the microbial community in these niches. The biological availability of Fe is complicated by the presence of diverse organic ligands that bind to it. The high demand and low bioavailability of Fe mean that it is an abiotic stressor driving the evolution of microbial Fe-metabolism. We calculated rates of non-synonymous mutations for a set of genes that were discriminating between the above mentioned niches which were distinct with respect to temperature, dFe concentrations, or Coastal and Open Ocean location, and inferred that genes that exhibited higher rates of non-synonymous mutations were the ones involved in non-specific uptake of Fe bound to diverse ligands. This indicated that in the highly diffusive, oligotrophic open ocean marine environment possession of Fe uptake strategies with broad specificities provides a competitive edge to the microorganisms.

## Conflict of Interest Statement

The authors declare that the research was conducted in the absence of any commercial or financial relationships that could be construed as a potential conflict of interest.

## Supplementary Material

The Supplementary Material for this article can be found online at http://www.frontiersin.org/Microbiological_Chemistry/10.3389/fmicb.2012.00362/abstract

## Footnotes

^1^http://www.moore.org/microgenome/

^2^http://www.genomics.ceh.ac.uk/GeneSwytch/Tools.html

^3^http://metagenomics.anl.gov/
